# Does a biomedical research centre affect patient care in local hospitals?

**DOI:** 10.1186/s12961-016-0163-7

**Published:** 2017-01-21

**Authors:** Catherine A Lichten, Grace Marsden, Alexandra Pollitt, Vasiliki Kiparoglou, Keith M Channon, Jon Sussex

**Affiliations:** 1 RAND Europe, Westbrook Centre, Milton Road, Cambridge, CB4 1YG United Kingdom; 2 Office of Health Economics, Southside 7th floor, 105 Victoria Street, London, SW1E 6QT United Kingdom; 3 The Policy Institute, King’s College London, London, WC2B 6LE United Kingdom; 4 NIHR Oxford Biomedical Research Centre, Oxford University Hospitals NHS Foundation Trust, John Radcliffe Hospital, Oxford, OX3 9DU United Kingdom

**Keywords:** Research activity, Hospitals, Patient care, Impact

## Abstract

**Background:**

Biomedical research can have impacts on patient care at research-active hospitals. We qualitatively evaluated the impact of the Oxford Biomedical Research Centre (Oxford BRC), a university-hospital partnership, on the effectiveness and efficiency of healthcare in local hospitals. Effectiveness and efficiency are conceptualised in terms of impacts perceived by clinicians on the quality, quantity and costs of patient care they deliver.

**Methods:**

First, we reviewed documentation from Oxford BRC and literature on the impact of research activity on patient care. Second, we interviewed leaders of the Oxford BRC’s research to identify the direct and indirect impacts they expected their activity would have on local hospitals. Third, this information was used to inform interviews with senior clinicians responsible for patient care at Oxford’s acute hospitals to discover what impacts they observed from research generally and from Oxford BRC’s research work specifically. We compared and contrasted the results from the two sets of interviews using a qualitative approach. Finally, we identified themes emerging from the senior clinicians’ responses, and compared them with an existing taxonomy of mechanisms through which quality of healthcare may be affected in research-active settings.

**Results:**

We were able to interview 17 research leaders at the Oxford BRC and 19 senior clinicians at Oxford’s acute hospitals. The research leaders identified a wide range of beneficial impacts that they expected might be felt at local hospitals as a result of their research activity. They expected the impact of their research activity on patient care to be generally positive. The senior clinicians responsible for patient care at those hospitals presented a more mixed picture, identifying many positive impacts, but also a smaller number of negative impacts, from research activity, including that of the Oxford BRC. We found the existing taxonomy of benefit types to be helpful in organising the findings, and propose modifications to further improve its usefulness.

**Conclusions:**

Impacts from research activity on the effectiveness and efficiency of patient care at the local acute hospitals, as perceived by senior clinicians, were more often beneficial than harmful. The Oxford BRC contributed to those impacts.

**Electronic supplementary material:**

The online version of this article (doi:10.1186/s12961-016-0163-7) contains supplementary material, which is available to authorized users.

## Background

Improving the translation of scientific discoveries into health benefits for patients and the population in general has long been an aim of government policy in the United Kingdom and internationally. According to the Cooksey review of United Kingdom health research funding [1], there are two gaps in the translation of health research into practice: the first is in translating basic and clinical research into the development of new products, technologies and approaches to the treatment of illness and health, and the second is in implementing these products, technologies and service approaches in clinical practice.

In England, direct manifestations of the policy aim to improve research translation have been the creation of the National Institute of Health Research (NIHR) in April 2007, supported by the United Kingdom Government’s Department of Health, and subsequent funding of ‘Biomedical Research Centres’ (BRCs) as well as Biomedical Research Units, Collaborations for Leadership in Applied Health Research and Care, Academic Health Sciences Centres (AHSCs), and Academic Health Sciences Networks. Each BRC is a partnership between a university and a National Health Service (NHS) hospital organisation. A major explicit aim of BRCs is to “*translate advances in biomedical research into benefits for patients*” [2] and thus address the first gap identified by the Cooksey Review.

Outside the United Kingdom, several countries, including Canada, the Netherlands, Singapore and the United States of America, have developed collaborative AHSC models linking academic institutions with healthcare delivery systems [3]. Among other international efforts to promote translation, in the Unites States in 2006, the National Institutes of Health introduced the Clinical and Translation Science Awards Program to address two main aspects of research translation: the advancement of discoveries from basic and preclinical research into human studies, and the dissemination and adoption of best practices [4]. Housed in the National Center for Advancing Translational Sciences (established in 2011), the programme funds a network of sites across the United States that provide support in a range of relevant areas including infrastructure, regulatory support, patient recruitment, researcher training, and statistics expertise.

NIHR funding of NHS hospital–university partnerships through the BRCs aims to bring together discovery science capabilities and strengths (typically based in the university partner) with clinical research platforms, personnel and patients (typically based in the NHS hospital partner). In this way, the NIHR BRCs form an important component of the United Kingdom’s AHSCs, aligning with other major centres based on partnerships between hospital and university organisations internationally.

Biomedical research can have substantial and varied impacts on patient care and on society more widely [5, 6]. A perceptions audit of senior hospital executives and medical school deans involved in the BRC scheme carried out 18 months after the establishment of the BRCs highlighted their role in building capacity in translational research and strengthening collaborative relationships between the NHS, academia, industry and other stakeholders [7]. That study also noted that it was too early to measure benefits (or otherwise) for patients at the time. Several years on, an initial evaluation of the impacts on patient care has become possible.

We report here our qualitative evaluation of the impact of the NIHR Oxford Biomedical Research Centre (hereafter Oxford BRC) on the effectiveness and efficiency of healthcare provided in local NHS hospitals. Effectiveness and efficiency are conceptualised in our study in terms of impacts perceived by clinicians on the quality and quantity of patient care they deliver, alongside their perceptions of the impacts on the costs of (resources used by) doing so.

Oxford BRC was one of the five large ‘comprehensive’ BRCs in England that have been funded by the NIHR since April 2007 (there are, additionally, several other BRCs focused on narrower subsets of biomedical research). The NIHR Oxford BRC is a partnership between the University of Oxford and the Oxford University Hospitals NHS Foundation Trust (hereafter OUH), which operates four publicly owned acute teaching hospitals – three large tertiary centre hospitals in Oxford and a small general acute hospital in the town of Banbury. The University of Oxford is a major university and conducts a large amount of biomedical research, which provides the basis for the Oxford BRC. OUH provides the full range of emergency and non-emergency acute secondary hospital services for the city of Oxford and the surrounding areas of Oxfordshire, along with tertiary hospital services for a much wider region across South-Central England. The University of Oxford and OUH are partners in the Oxford Academic Health Science Centre, a partnership that also includes Oxford Brookes University and Oxford Health NHS Foundation Trust, a neighbouring NHS hospital that provides clinical services in mental health and community care, and hosts an NIHR BRC focussing on mental health. Oxford BRC conducts translational research within 14 research themes (RTs) and has also established seven ‘Working Groups’ (WGs) to address strategic priorities that cross multiple RTs.

There is a growing literature on the effect on the performance of healthcare providers if their clinical staff are engaged in research. When research takes place in a clinical setting, this may affect the research itself – for example, by facilitating more effective or efficient clinical trials [8] or in relation to the aspects highlighted by Marjanovic et al. [7], namely improving research relevance and prioritisation of research resources, building capacity in translational research; and strengthening collaborative relationships between the NHS, academia, industry and other stakeholders. It may also have an effect on the patient care provided within that institution [9]. Research may have direct impacts by bringing about changes to healthcare, like a new diagnostic tool or treatment or a reorganisation of processes for delivering care. It may also have indirect impacts through, for example, changes to provision of equipment, training, or changes in attitudes to research among staff.

While evidence has not been unanimous on the existence and nature of these impacts on patient care, Hanney et al.’s [10] major review of the literature concluded that there is indeed an overall positive association between the engagement of individuals and healthcare organisations in research and levels of healthcare performance. The authors have helpfully developed a taxonomy of five types of mechanism through which such improvements might occur, set out in Box 1. These mechanisms are in addition to any benefits derived directly by patients as a result of their own participation in clinical trials. We have compared our findings with this taxonomy of mechanisms, which for brevity we refer to as ‘the Hanney framework’ from here on.

More recently, Ozdemir et al. [11] have reported positive and statistically significant associations implying that research-active NHS hospitals in England have lower risk-adjusted mortality for acute admissions, which persisted after adjustment for staffing and other structural factors. More recently still, Harding et al. [12] have presented a systematic review of literature specifically focused on associations between research culture in healthcare organisations and their performance as care providers. They find eight studies satisfying their criteria (including [11] and overlapping with the broader review by Hanney et al. [10]) and conclude that a “*stronger research culture appears to be associated with benefits to patients, staff and the organisation*”.

We note that the original definition of ‘absorptive capacity’, coined by Cohen and Levinthal [13], was “*the ability of a firm to recognise the value of new, external information, assimilate it, and apply it*” and which was suggested to be “*largely a function of the firm’s level of prior related knowledge*”. Following the lead of Hanney et al. [10], we interpret this as having a broad application that can be extended beyond existing knowledge (the main example by Cohen and Levinthal) to existing infrastructure.

## Methods

Our research design is qualitative, which inevitably means that we are not able to quantify any impacts found. We considered that a qualitative approach was essential to explore the subtle nature and extent of any possible impacts amid the numerous confounding factors that influence available measures of care outcomes, and costs of care, at OUH.

To assess the impacts of the Oxford BRC on the patient care provided at the OUH hospitals, we used a multi-phase approach. First, we reviewed documentation provided by the Oxford BRC (including its own reports and other documentation concerning its research) and relevant literature on evaluating the impact of research activity on patient care. This work was conducted to inform our approach in the subsequent phase of this study, rather than to provide an analysis of all the existing literature in this area.

Second, we spoke to representatives of the Oxford BRC (leaders of the 14 RTs and 3 of the WGs). The purpose of these interviews was to identify the impacts that the RT and WG leaders in Oxford BRC expected would have resulted from their RT/WG work, including both direct and indirect impacts. This information was used to provide background and structure to a subsequent set of interviews with senior OUH clinicians.

Third, we interviewed senior OUH clinicians responsible for patient care (the Directors of the hospital’s clinical Divisions and Directorates) to find out what impacts they had observed and to test whether the impacts that the Oxford BRC RT/WG leads expected were observed by the clinicians. We targeted our interviews at this level of seniority, within the constraints of budget and timescale, in order to cover all areas of patient care. It is possible that different impacts of research activity might be felt, and to different degrees, at different levels. The OUH clinicians were asked first about the effects of research activity generally in their Division or Directorate, and were then asked about Oxford BRC research specifically. We then identified a set of themes from the responses to these interviews.

Fourth, we conducted a ‘cross analysis’ in which we compared and contrasted the results from the two sets of interviews.

Finally, we compared the themes which had been identified from the senior clinician interviews with the mechanisms outlined by Hanney et al. [10] (Box 1). The purpose of this was to test the list of suggested mechanisms against our results and not to force our results into the categories suggested by Hanney et al.

### Oxford BRC Research Theme/Working Group (RT/WG) lead interviews

The Oxford BRC’s RT/WG leads were informed about the study and purpose of the proposed interviews by email and in person (by JS) at one of their monthly meetings. They were then invited by email to participate in the interviews. Invitations were sent to all 14 of the Oxford BRC RT leads plus 3 WG leads. The latter were interviewed at the recommendation of the Oxford BRC manager on the basis that their WG work may also have brought about observable impacts in the OUH. All 17 of the invited RT/WGs, listed in Box 2, provided a representative to participate in the interviews.

The interview protocol (Additional file 1) was sent to the interviewees 2–3 days in advance of the interview. The protocol included a reminder of the project goals and activities, a note on confidentiality, and the questions which would be used to guide the discussion.

Seventeen interviews were conducted, by telephone, by one researcher in each case (CL, GM or JS), from September to November 2015. The interviews each lasted 45–60 minutes and were audio recorded with consent of the interviewees. After each interview, the notes were shared with the interviewees and their co- and/or deputy-leads to allow for amendments or corrections. The notes were detailed and covered the majority of the conversation, but were not full transcripts. For the majority of the interviews, there was one interviewee present, namely the RT lead or WG lead or one of the co-leads. In two cases, the interviewee was a colleague nominated by the lead; in four cases, the interviewee was joined by a colleague from the RT/WG. The interviews were semi-structured so as to elicit comparable answers across the RT/WGs to common questions, while also encouraging open responses discussing the types of impacts and the pathways by which they are achieved.

The questions were designed to explore the expected direct and indirect impacts of specific research projects that have been part of each theme, with the focus primarily on impacts felt at OUH. The section on indirect effects specifically highlighted collaboration and interaction, absorptive capacity, infrastructure and human capital, action and participatory research, and economic impacts (note that economic impacts are not the focus of this paper; responses to these questions were used to inform a parallel research project to be reported separately).

Interviews were analysed systematically. First, the researcher who had conducted the interview entered the results concisely into an Excel spreadsheet, so that the outcomes of all 17 interviews could be seen together. The results were grouped into ‘key areas’ with a distinction made between direct and indirect impacts. Where relevant, we aligned the ‘key areas’ with the categories of mechanisms listed by Hanney et al. [10], but we did not force the responses into the categories where they are not a good fit. All researchers who had conducted the RT/WG interviews (CL, GM and JS) reviewed the overall spreadsheet and identified where clarification was needed, which the relevant interviewer then actioned. In total, across the 17 interviews, 44 projects or clinical services enabled by Oxford BRC-related projects were identified by RT/WG leads as expected to have a direct impact on the OUH. They are listed Table 1 in the results section. The study team identified, for each item on the list, which clinical areas within the OUH might be expected to have encountered it.

**Table 1:** Summary of Oxford Biomedical Research Centre (BRC) projects that Research theme/Working group leads indicated had potential impacts on patient care at Oxford University Hospitals NHS Foundation Trust (OUH)

BRC Research theme/Working group	Oxford BRC projects with potential impact on patient care	Potential impact on patient care at OUH
Biomedical Informatics and Technology	Gestational diabetes smartphone application	Processes and/or service organisation
	New alerting score based on patients’ vital signs (CALMS-3 Study – Computer Alerting Monitoring System)	Processes and/or service organisation
	Maintenance of database of samples and consent management for Oxford Biobank	Processes and/or service organisation
	System for Electronic Notes Documentation (SEND) project (electronic track and trigger and patient alerting)	Processes and/or service organisation
Blood	Training and hiring staff and improving infrastructure for clinical programme to do stem cell transplants and run more clinical trials in this area	Treatments
Cancer	Multi-gene testing service with rapid (1 week) turnaround	Diagnostics, testing, screening
	BRC-funded clinical research posts specialising in sarcoma, gynaecological cancers and melanoma	Staffing for specialist services
Cardiovascular	MRI diagnostic tools for patients with intermittent angina	Diagnostics, testing, screening
Clinical Informatics	Upgrade for cancer informatics in OUH (enabled by Genomic Medicine Centre (GMC) designation)	Diagnostics, testing, screening
	Molecular diagnostics and genome sequencing services (enabled by GMC designation)	Diagnostics, testing, screening
	Development of integrated logical record for each cancer patient (in progress)	Processes and/or service organisation
	True colours technology for mental health patients across Oxfordshire to aid self-management	Processes and/or service organisation
	Streamlining management of notes/records from multidisciplinary cancer meetings (in progress)	Processes and/or service organisation
	Clinical decision support dialogue to make blood orders more efficient	Processes and/or service organisation
Dementia and Cerebrovascular Disease	New screening process for acute confusion in medical admissions	Diagnostics, testing, screening
	Fast track carotid surgery	Treatments
	Telemetric home blood pressure monitoring	Processes and/or service organisation
	The first transient ischaemic attack (TIA) and minor stroke clinic in the United Kingdom	Processes and/or service organisation
Diabetes	Diagnostic programme to identify patients with a genetic basis for their diabetes	Diagnostics, testing, screening
	Pancreatic islet extraction unit	Treatments
	Pancreatic transplants	Treatments
Functional Neuroscience and Imaging	Imaging protocols (epilepsy)	Diagnostics, testing, screening
	Functional neurosurgery team (part of the BRC) provides a clinical service, including pain deep brain stimulation	Treatments
	Specialist clinic in epilepsy, Parkinson’s disease, motor neurone disease, supported by BRC-funded research fellows	Staffing for specialist services
Genomic Medicine	Targeted next generation sequencing	Diagnostics, testing, screening
	Whole exome or whole genome sequencing	Diagnostics, testing, screening
Immunology and Inflammation	Non-invasive tests for liver disease	Diagnostics, testing, screening
	Patient stratification for biologic therapies	Diagnostics, testing, screening
	Tests to diagnose encephalitis (brain inflammation)	Diagnostics, testing, screening
	Trials on treatment of inflammation in eczema	Treatments
Infection	Work linking care to outcomes with big data approaches	Processes and/or service organisation
	Genomic testing to support infection monitoring (provided informally)	Processes and/or service organisation
Molecular Diagnostics	Oxford designated as GMC for Genomics England’s 100,000 Genomes Project	Diagnostics, testing, screening
	Multi-gene testing for cancer patients	Diagnostics, testing, screening
	Diagnostic tests for children with rare blood conditions	Diagnostics, testing, screening
	Response prediction in leukaemia	Diagnostics, testing, screening
Prevention and Population Care	The first TIA and minor stroke clinic in the United Kingdom	Processes and/or service organisation
Surgical Innovation and Evaluation	Chemotherapy (device targeted therapy): liposomes developed to carry the treatment to the organ of interest	Treatments
	Image-guided surgery using fluorescents	Treatments
	Organ transplantation/reconditioning	Treatments
	Restoring vision through an electronic implant	Treatments
	Restoring vision through gene therapy	Treatments
Training and Education	No projects identified	
Translational Physiology	No projects identified	
Vaccines	Respiratory syncytial virus, trialling a new vaccine	Treatments (national)
	Meningitis, looking at trials of schedules for baby vaccines, booster doses, meningitis B vaccine trials	Processes and/or service organisation (national)
Multiple research themes/Working groups	Better infrastructure for clinical trials and other studies (e.g. better pharmacovigilance, biobanking)	Processes and/or service organisation

### OUH senior clinician interviews

The OUH is organised into five Divisions, with two to five Clinical Directorates per Division, giving a total of 18 Clinical Directorates. All 23 of the divisional directors and clinical directors were invited to participate in the interviews and all 23 were clinicians. They were informed about the study and the purpose of the interviews via an email from the office of the Oxford BRC Director, who was also the OUH Director of Research. The email explained that the study had been commissioned by Oxford BRC to understand the extent of its impact and to explore how it could operate more effectively in the future.

Nineteen of the 23 clinical heads of Divisions/Directorates participated in the interviews, as did three of the five divisional heads and 16 of the 18 heads of the Directorates which fall within those Divisions. Four declined to be interviewed (two divisional directors and two clinical directors; Box 3). One interviewee changed roles from clinical director to divisional director during the course of the study.

Two of the divisional directors were also Oxford BRC RT leads; they were interviewed again for this phase of the study, and asked to discuss their observations from the perspective of their roles as divisional directors. These interviewees’ status as RT/divisional director hybrids is likely to have impacted their responses (i.e. the responses are based not only on their experiences as divisional directors but naturally influenced by the research that they are involved in) and we have therefore identified this within the results section where it may affect the findings.

The interviewees were sent a reminder by email 2 or 3 days ahead of the interview. The email reiterated the purpose of the interview, noted that interviewees were not expected to prepare for the interview or be familiar with the Oxford BRC, and included the list of indirect impacts that would be asked about (Box 1). The list was sent to familiarise interviewees with the notion of indirect impacts, so that they did not unnecessarily limit their thinking solely to direct impacts. (The interviews were to determine whether and how Oxford BRC research projects were considered by clinicians to be impacting patient care at their hospital, not to test clinicians’ familiarity with the Hanney framework). No mention was made of any individual Oxford BRC research projects at this stage.

All of the clinician interviews were carried out face-to-face at the OUH hospitals in Oxford (the interviewers were AP, CL, GM and JS) in December 2015 and January 2016. At each interview, one researcher conducted the interview and another took notes. The notes were based on concurrent note-taking (rather than audio taping) and were detailed but were not full transcripts. The notes were shared with the interviewees shortly after the interview to allow for corrections or amendments. For the majority of the interviews, only the invited interviewee was present; for two interviews, a colleague of the interviewee joined and contributed.

The interviews followed a structured protocol (Additional file 2) to ensure comparability of responses across interviews. At the start of each interview, the interviewee was reminded about the purpose of the interview. The questions about the impact of research activity were divided into three parts:

#### Part 1

Observations about research as it relates to the Directorate’s/Division’s clinical work. These questions explored the role of research in the Directorate/Division and OUH staff involvement with research. It included a series of 10 questions covering the types of indirect impacts outlined in the Hanney Framework (Box 1).

#### Part 2

Observations about Oxford BRC-specific research activities. These questions focused on the impacts of Oxford BRC research specifically and the role of the Oxford BRC.

#### Part 3

Specific examples of Oxford BRC projects. Interviewees were first asked, without prompting, to name any Oxford BRC projects they were aware of and that had affected patient care in their Directorate/Division. Once no further projects were forthcoming, each interviewee was then prompted with a set of projects. These were items (usually 4–5) from the list of 44 examples of direct impacts given by the Oxford BRC RT/WG leads that the researchers considered the interviewee may be familiar with because they had a potential impact on the work of that Directorate/Division.

The interviewees’ responses were systematically coded and analysed. First, one researcher who had participated in the interview (AP, CL, GM or JS) entered relevant comments from each interview into a spreadsheet matrix (rows = interviewees, columns = interview questions). These entries were then reviewed by the second researcher present for each interview (AP, CL or GM) and any differences of interpretation were discussed and resolved. Then, for each question in turn, a researcher (CL or GM) compared the responses across the 19 interviews, assessing the different types of response and quantifying how many respondents gave each type of response. These summaries were then checked by another researcher (JS). Finally, the whole research team discussed the results and emerging themes across questions, and agreed the final list of themes.

### Cross-analysis of results

To obtain a more complete picture of each of the themes identified from the analysis of the OUH clinician interviews data, we reviewed comments made by RT/WG interviewees that aligned with the themes we identified from the clinician interviews. We compared and contrasted the perspectives of the two sets of interviewees and summarise this analysis in the final results section (note that the RT/WG interviewees were not systematically asked about the themes that arose in the interviews with clinicians, which took place after the RT/WG interviews).

## Results

### Results of the RT/WG leader interviews

Based on what they told us, we have grouped the RT/WG leads’ responses to the interview questions into the following main areas (note that some of them, but not all, correspond to mechanisms suggested by Hanney et al. [10] (Box 1):

- Direct impacts of Oxford BRC research on patient care
- Indirect impacts:
  - ○ Absorptive capacity: infrastructure
  - ○ Absorptive capacity: developing human capital
  - ○ Improvements related to clinical trials (enabling more trials or closer monitoring and support)
  - ○ Collaborative working and participatory research

We report here our findings from what was said in the interviews. They should not be taken to represent a comprehensive account of all BRC projects (as there are over 400 of them) or all possible impacts of those projects.

#### Direct impacts

Interviewees explained that more than half of the Oxford BRC budget is used to pay staff salaries, while the rest covers the costs of consumables and other research-related activities. The direct impacts described were thus related to projects carried out by staff who were at least partly Oxford BRC-funded, or were related to other activities or improvements in research capacity that were enabled by the Oxford BRC’s support.

The RT/WG interviewees identified 44 projects or clinical services enabled by Oxford BRC-related projects that they thought had directly impacted the care of OUH patients and/or the organisation of clinical services in the OUH. These Oxford BRC projects are listed in Table 1. To better understand the types of impacts occurring, we have grouped the projects according to four areas of primary impact, with each project falling within just one of the four headings:

- Diagnostics, testing or patient screening
- Treatments available
- Processes for delivering patient care and/or organising hospital services
- Staffing for specialist services

Among the total of 44 projects, 16 had impacts related to diagnostics, testing or patient screening, 12 on treatments available, 14 were related to processes for delivering patient care and/or organising hospital services, and two had impacts on staffing for specialist services (Table 1). Many of the RT/WG interviewees also described ongoing projects they thought were likely to have future impacts on OUH; however, the present study focuses on existing impacts, so these are not listed.

An example of a development that affected the diagnosis or screening of patients is that Oxford was designated as one of the 11 NHS Genomic Medicine Centres (GMCs) in 2014. One interviewee explained that the Oxford BRC had played an important role in enabling Oxford to achieve this designation, and that it had led to a major upgrade for cancer informatics services as well as boosting OUH’s molecular diagnostic and genome sequencing services. Another interviewee had observed that genetic testing services had improved, with the turnaround time for results shortened.

A project that impacted how care is organised and delivered was the System for Electronic Notes Documentation (SEND) project, an electronic system for patient monitoring and staff alerting that was developed with Oxford BRC support and was being rolled out across OUH’s four hospitals at the time of the interview. The Oxford BRC was also seen to have impacted on specialist clinics in epilepsy, Parkinson’s disease and motor neurone disease, which are supported by Oxford BRC-funded research fellows.

### Indirect impacts

#### Absorptive capacity: infrastructure

Interviewees reported that Oxford BRC provides research infrastructure support to the OUH in two ways and thereby has a positive impact on patient care: it enables more clinical trials and other research studies to be carried out (discussed in a separate section below), and it contributes indirectly to the acquisition of equipment used for research. One interviewee reported that the presence of staff from the Oxford BRC’s WGs on Clinical Informatics and Molecular Diagnostics had enabled OUH to demonstrate that they had the necessary capabilities to become a Genomic Medicine Centre, which in turn enables better care at OUH.

Other forms of Oxford BRC-related improvements to infrastructure at OUH that were cited were better biobanking, including a tissues database and consent management system (cited by three interviewees) and improved pharmacovigilance and pharmacy support (cited by two interviewees). Overall, the idea that the Oxford BRC is an important enabler came across strongly in the interviews with RT/WG leads. As one RT lead said: “*The BRC is highly enabling to* [our work in applying state of the art techniques to answer pressing problems] *because it gives us resources, it gives us focus*”.

Eight RT/WG interviewees explained that, whilst BRC funding by NIHR is not provided for capital spending, the Oxford BRC helps contribute to legitimate revenue costs and RT leads are asked to source capital expenditure from other funding sources. For example, in the Genomic Medicine RT, all of the sequencing technology platforms were purchased with funds from research grants. In this case, the equipment subsequently became available for non-research use by OUH staff. Another interviewee suggested that the Oxford BRC may have helped the Oxford Centre for Functional MRI of the Brain, a neuroimaging research facility, to attract some of its funding.

Interviewees reported that the Oxford BRC also contributes to the running costs of some facilities at OUH, with the following examples each being mentioned once: the Acute Vascular Imaging Centre (AVIC), an advanced clinical research facility that has an MRI scanner conjoined to an angiography suite; the Oxford Centre for Diabetes Endocrinology and Metabolism (OCDEM) pancreatic islet extraction unit; and the Oxford Clinical Biomanufacturing facility, which produces products for early-phase clinical trials. The Oxford BRC has also provided funding for consumables and smaller scale equipment such as blood pressure monitors. Through one RT, the Oxford BRC covered the cost for patients to receive genetic tests that assess whether there is a genetic basis for their diabetes. In another RT, the Oxford BRC funded scans that were part of a larger project on acute myocardial infarction. Interviewees said it would be difficult to purchase these services, pieces of equipment and consumables without Oxford BRC funding.

#### Absorptive capacity: developing human capital

The main mechanism through which the Oxford BRC supports human capital development at OUH is funding for staff to do research. This includes paying for medical staff to reduce their clinical hours and devote some time to research, funding research fellows and trainees who also do clinical work, and paying for non-medical staff such as nurses to do, and contribute to, research. Six interviewees referred to the importance of Oxford BRC-funded research nurses for doing clinical research, although one interviewee also noted as a downside that nurses who move into wholly research positions do not usually go back to working on the wards, which can imply a loss of high quality staff from patient care. Also mentioned was Oxford BRC support for radiographers, genetic counsellors, statisticians, pharmacists, project managers with expertise in clinical governance, and database staff. Five interviewees mentioned that it would not have been possible to engage these staff without the Oxford BRC.

The opportunities to have time ‘bought out’ or ‘protected’ for research and the increased involvement of clinical staff in research were seen by several RT/WG interviewees as having had positive effects on recruitment and organisational culture at OUH. Six RT/WG interviewees expressed the view that the research opportunities offered by the Oxford BRC help attract and retain high-quality staff, and one added that the Oxford BRC’s reputation also attracts staff (both medical and non-medical). One interviewee explained that the Oxford BRC has “*provided more headroom*” for busy staff to think about and do research by introducing additional funding and increasing time available for research and implementation of new programmes. Another said that the Oxford BRC had “*transformed*” research in intensive care by helping to bring in more researchers, including a clinical lecturer, clinical academic fellows and academic foundation doctors.

Some of the interviewees felt that, by allowing more staff to do research, the Oxford BRC is helping bring about a cultural change through which research is gaining traction and becoming more important in the day to day work of the OUH. They described a positive feedback loop, where OUH employees’ increased involvement in research leads to more research interactions, and a change in attitudes toward research. Some reported that research became more deeply embedded within the clinical departments. However, this view was not shared by all of the RT/WG interviewees; one explicitly said that no cultural change had occurred and that “*research is still seen as being for researchers, not everyone*”.

In addition to the Oxford BRC’s role in supporting the staff who do research (including research fellows, consultants and non-medical staff), interviewees described how the Oxford BRC helps to develop human capital by enabling medical trainees to obtain research experience and formal research training. Representatives of the Research Education and Training WG cited Oxford BRC funding for doctorates, clinical fellowship schemes, training bursaries and other courses.

Five interviewees highlighted examples of training taking place within their RTs. This included opportunities for junior doctors to receive research training as well as opportunities for staff to learn new techniques, access new technologies, and be “*exposed to different ways of thinking about disease*”. One interviewee emphasised that junior doctors are encouraged to attend weekly research meetings and get involved in research, so that they could become “*research-trained doctors, not ordinary doctors*”. This interviewee commented that, without being able to have some of their time protected for research, NHS trainees cannot step off their training pathway and obtain research experience. Another interviewee reported that a cohort of haematologists and PhD fellows in haematology had been trained with support from the Oxford BRC and other funding sources.

Some formal training opportunities target non-medical staff. For instance, an interviewee reported that within their RT, nursing staff have been on Oxford BRC-funded training courses related to treatment of stroke and hypertension patients. Other interviewees explained that an orientation pack has been developed with Oxford BRC funding for people entering new jobs as research nurses, and that other Oxford BRC-funded training opportunities have included courses on how to prepare patients for trials, laboratory skills, research methodology, design and statistics, and management.

#### Improvements related to clinical trials

One interviewee explained that the Oxford BRC supports senior staff who have critical knowledge of how to set up clinical trials and how to write protocols, contracts and ethics submissions. Four RT/WG interviewees described how the Oxford BRC had helped to increase clinical trials capacity and enabled more trials to take place. For example, one interviewee explained that, in their field, clinical research had previously been undertaken on a relatively small scale and had grown with the Oxford BRC so that now approximately 2000 patients are recruited for studies each year. Another interviewee said that they used to run just two or three clinical trials at a time but now they run more than 60, and that the change was enabled in part by the establishment of a clinical trials unit run by staff who receive funding from a mix of sources including the Oxford BRC. Two interviewees felt that the Oxford BRC had a particular impact on improving the quality and scope of early-phase trials done in the OUH.

#### Collaborative working and participatory research

A commonly stated view across the RT/WG leads was that the Oxford BRC had led to more active collaboration between OUH clinical staff and academic researchers. One interviewee described this collaboration as an important part of the ‘ethos’ of their RT, while another observed that Oxford BRC funding is dependent on collaboration and had thus brought about a different way of working. Others commented that the Oxford BRC enables collaboration because more staff hold joint appointments with both the University of Oxford and the OUH. Two interviewees explicitly stated that the introduction of these joint appointments together with the Oxford BRC’s research nurses had “*bridged the gap*” between the University and the OUH.

In addition, two interviewees reported that the Oxford BRC has worked hard to align its research with the OUH’s strategic priorities. Two interviewees explained that both the University and the OUH benefit from the increased collaboration, as the academics gain the opportunity to collaborate with clinical personnel and work with patients, while the NHS gains access to research funding and research capabilities. They emphasised that the interaction helps leverage more research funding (including grants and industry collaborations) into both the OUH and the University.

RT/WG interviewees also described how increased participation by hospital staff had made research more clinically relevant and raised the profile of research in the OUH. Three interviewees explained that research relevance was increased as researchers were brought closer to the clinical setting and would thus ask more clinically relevant questions, with one saying that researchers with a “*clinical footprint*” are more likely to undertake research that is clinically relevant. Another interviewee explained that the Oxford BRC seeks to do translatable, economically viable research, and so Oxford BRC researchers are expected to deliver projects in line with these aims. One interviewee described how the SEND project had evolved through collaboration between bioengineers and full-time clinicians. They predicted that as SEND was rolled out, clinicians who use it would be interested in getting more actively involved with it – both by providing suggestions to further develop it and by making use of the data it collects. Interviewees also noted that research that has been demonstrated to work in a clinical setting has more credibility, and that staff become more interested in research after seeing its tangible benefits. One interviewee added that they felt the Oxford BRC had dramatically improved the perception of research within the OUH: “*It is undoubtedly the case that the profile of what we do, particularly among non-medical NHS staff, has been greatly enhanced by the BRC*”. However, the same interviewee acknowledged there is still some way to go in making the link between research and clinical application seamless.

Six interviewees felt that the Oxford BRC has increased the profile of patient engagement in research or identified positive achievements of the Oxford BRC’s patient and public involvement (PPI) work. However, the remaining respondents were either unsure whether there had been an impact in this area or did not discuss Oxford BRC-related PPI activities. Of those who did consider that there had been an impact, one highlighted that training is run with the Oxford BRC’s PPI WG to raise awareness among the researchers; and three highlighted that the Oxford BRC has held popular Open Days to showcase its work to patients and public. Interviewees described examples of how the Oxford BRC’s PPI work programme is having a visible impact. Two described examples of how patients had been significantly involved in research design; two others mentioned that researchers are asked to write lay summaries of their work with help from the PPI working group; and another noted that an Oxford BRC research nurse sits on the hospital’s patient forum.

### Results of the OUH senior clinician interviews

The interviews with the RT/WG leads, supported by our review of Oxford BRC documents, provided an overview of the RT/WG leads’ research activity and where they would expect OUH staff may have felt an impact from that research. It is to be expected that the RT/WG leads would tend to a positive view of the impact of the Oxford BRC on healthcare in OUH although limitations to that impact were also recognised. While the purpose of the interviews with the RT/WG leads was to determine the potential scope of impacts, the purpose of the interviews with senior clinicians responsible for patient care at OUH was to determine to what extent such impacts were observed in practice. In the interviews with clinical heads of Directorates and Divisions at OUH, we first discussed the impact of research activity at OUH on patient care. We then asked more specifically about the contribution of the Oxford BRC to that impact. When arranging the interviews with OUH senior clinicians, the request for interviews explicitly stated that the study had been commissioned by Oxford BRC. At the interviews themselves, all 19 interviewees (100%) confirmed that they were aware of the Oxford BRC. More informatively, nine (47%) of them went further to say that they were familiar with, or work directly with, the Oxford BRC.

The results from these interviews are discussed in the following pages in four parts: first, we outline the themes which were identified from the interviewees’ responses; second, we provide a summary of interviewees’ awareness of specific Oxford BRC-related projects; third, we discuss the impact of the Oxford BRC on collaboration between OUH staff and others, including University researchers; and finally, we analyse the responses in the context of the Hanney framework.

#### Themes from the interviews with OUH clinicians

Our thematic analysis of the full set of interview notes led us to identify 10 themes emerging from the discussions with senior clinicians at OUH:

1. Research activity
2. Formalisation of research roles
3. Communication and awareness of research
4. Reputation
5. Staff recruitment and retention
6. Patient benefits from staff involvement in research
7. Access to infrastructure
8. Novel treatments and technologies
9. Attitudes to research
10. Collaboration

Note that many of these themes link together and overlap with each other. For example, it is likely that reputation (Theme 4) aids staff recruitment and retention (Theme 5), and that attitudes to research (Theme 9) are influenced by communication and awareness (Theme 3). The themes that emerged from the interviews with OUH senior clinicians correspond to varying degrees with the mechanisms in the Hanney framework.

Each theme is discussed in turn below, including how it relates to the Hanney framework. Then, the combined picture across all themes is summarised. The majority of the themes point to some positive changes that have been achieved, but some also highlight negative consequences.

##### Theme 1 – Research activity

Fifteen of the 19 interviewees (79%) stated that research does affect the NHS work (i.e. patient care) done within their Division or Directorate of the OUH, either positively or negatively or both. One of these respondents elaborated that: “*all patients are considered research patients by default*”. An additional three interviewees (16%) gave mixed responses, indicating that, whilst research is undertaken, it is not clear whether, or how far, this influences clinical work. Three respondents added that the amount that research affects the NHS work done varies depending on the clinical area. Only one interviewee (5%) said that research had no effect on patient care within their Directorate, which was a clinical support service. Taking all of these responses together indicates overall that there is the potential for substantial impact from research activity on OUH’s patient care services. The extent to which this impact was described as beneficial or otherwise is set out in the following sections, covering the other nine themes that emerged from the interviews with clinicians.

Interviewees were also asked to what extent clinical staff are involved in research and their responses indicate that this varies significantly. Seven interviewees (37%) indicated that they have many research-active colleagues, and a further five interviewees (26%) (one of whom was one of the divisional directors who is also an RT lead) indicated that all staff contribute to research as part of their regular work, e.g. CT scans or supplying medicines from the hospital pharmacy. Five respondents (26%) indicated that a minority have funding for research time, and four (21%) noted that there is higher involvement in research amongst consultants and other medical staff, compared to non-medical staff. Seven respondents (37%) noted that the involvement of clinical staff within research varies a lot between different staff members and between different units within the Directorates.

When asked whether clinical staff involvement in research had changed over time, 11 of the 19 interviewees (58%, including the two divisional directors who were also RT leads) felt that it had increased over time, one (5%) felt that it had decreased, and two (11%) felt that there had been no change. The remaining five responses (26%) offered no clear view.

Interviewees were aware of many Oxford BRC projects, and gave the impression that overall research activity continued to increase at OUH, at least in part due to the Oxford BRC. Thinking specifically about the Oxford BRC, 17 of the 19 OUH interviewees (89%) noted that the presence of the Oxford BRC has affected the research activities happening in their Directorate or Division. Of the two who said it has not, one works more closely with the separate NIHR Biomedical Research Unit based at the Nuffield Orthopaedic Centre (one of the four hospital sites within the OUH) than with the Oxford BRC, and the other suggested that research has no or little effect within their Division.

It is clear that Oxford BRC research plays a substantial role within the totality of research activity at OUH, but that involvement varies quite widely between Directorates/Divisions and interviewees were not always aware of what research was linked to the BRC. Eight interviewees (42%, including both of the divisional directors who were also RT leads) indicated that the proportion of research taking place within their Directorate or Division that is linked to the Oxford BRC in some way was large or a majority. An additional four (21%) indicated that the proportion was around 20–30%. Of the remaining seven, six (32%) felt unable to estimate the proportion, and one reported that no Oxford BRC research took place (this was the same respondent who indicated that research has no effect within their Division).

Interviewees were asked to consider the effects of the Oxford BRC hypothetically doubling in scale. The majority (14 respondents (74%), including the two divisional directors who were also RT leads) felt that this could be a positive change, but seven of these felt that such a change would need to be handled carefully because of the impacts research could have on staffing and physical space, for example. Specifically, two interviewees stated that the current allocation of funding is not spread evenly across the board; some areas already have a lot of research and may be saturated. Three interviewees noted that, in the words of one of them, “*any increase in funding would be less effective if it was put into areas which already have a lot of research underway*”.

When asked the opposite question, i.e. what would be the impact if hypothetically all of Oxford BRC’s funding were to be removed, only one respondent thought it would make no difference to their area of clinical work. All other respondents said the impact would be negative, but their answers ranged from describing small scale impacts to it being a “*disaster*”. One interviewee said: “*There would be a huge vacuum in activity because of how the BRC gets into the space between lab research and clinical work*”. Another commented that “*there would be a need to cut some clinical activities because the BRC funding sometimes supports activities that are clinical but for research purposes*”.

##### Theme 2 – Formalisation of research roles

Interviewees explained that increases in research activity have led to increases in research staff numbers. They said that involvement in research is now usually formalised with a clear distinction between individuals and, for each individual, between the different periods in the working week that are for research rather than patient care. Greater formalisation has the advantage, mentioned by several of the clinicians, of enabling research to be part of programmed activities rather than being squeezed into clinicians’ ‘spare time’. This was seen as allowing more ‘head space’ to think about research, with Oxford BRC and other research funding enabling staff to be recruited to cover for the time when clinicians work on research rather than provide patient care. The Hanney framework refers to this same set of issues as part of the third type of mechanism, as it lists ‘organisational mechanisms within healthcare systems’.

Thirteen of the 19 interviewees (68%) stated that research had changed how personnel are organised, three (16%) each said there had been no such change or were unsure or their answer was unclear. One interviewee mentioned that there are “*lots more research nurses*” around, and three others that clinical staff have given up sessions to do research. While this may facilitate more and better research, its impact on patient care is less clear. This latter point was echoed by three interviewees who described how clinical staff can be tempted away from a stressful clinical environment into less stressful research posts and how research can create a tension where some of the best staff are recruited to research posts full time and thereby taken away from patient care. This can be the case for nurses as well as medical staff.

When asked about factors that encourage staff to be involved in research, 6 of the 19 interviewees (32%) mentioned the opportunity to have protected time for research (either by the Oxford BRC or another source) as a key factor. In addition, when asked about barriers to research, 14 of the 19 (74%) mentioned a lack of time for research due to clinical workload as a key issue. This highlights that there is not always time or opportunity for someone to be involved in research, unless (some of) their time is formally allotted to research. When asked whether these enablers and barriers had changed over time, two interviewees specifically noted that now the only people doing research are those who are employed to do that, whereas in the past others had time for it too.

Some staff undertake both research and patient care activity. One interviewee commented that, whilst increases in staff are generally for research purposes, a key exception are clinical academics who are employed by the University. These clinical academics spend approximately half of their time working for OUH. In addition, some staff hold split posts (partly research, partly clinical), or have some of their clinical time ‘bought out’ (and therefore protected) for research. These split contracts have increased with the advent of the Oxford BRC. Another interviewee provided the example of some research fellows, who may be doing full time research, also contributing to on-call rotas at night, thereby reducing the burden of the night rota for clinical staff.

##### Theme 3 – Communication and awareness of research

This theme relates to internal and external communication about and awareness of current research activities, and the resulting effects on staff and patients. Hanney et al. [10] found from the literature that improving the absorptive capacity of staff who provide patient care is associated with improved quality of care. It is part of the first mechanism on their list by which research-active settings improve patient care. Communication and awareness of research contribute to that absorptive capacity.

When asked about factors that encourage staff involvement in research, four OUH senior clinician interviewees (21%) mentioned exposure to research, and two further interviewees (11%) mentioned awareness and opportunities. Four interviewees (21%) also stated that there have been improvements in raising awareness and providing more opportunities for research participation over time, one of whom stated that more of this is needed. Examples mentioned of ongoing activities to increase communication and awareness included seminars and discussions to disseminate research, and a board to display research posters from conferences.

The interviewees were also asked whether ongoing research has raised the profile of research within their Directorate or Division. Twelve of the 19 interviewees (63%) said yes, three (16%) said no, and four (21%) did not provide an answer. This ties back into the issue of awareness of research, and supports the previous comments that communication and awareness has increased, at least in some clinical areas.

When asked about barriers to research, four interviewees (21%) mentioned issues of poor awareness: two (11%) noted that staff do not know what research opportunities there are, and two more (11%) noted that the split between the NHS and the University means that many medical staff do not directly encounter research. The interviewees were also asked whether there are any changes which would enable more benefit to OUH from research being carried out, to which five respondents (26%) indicated that increasing awareness of research among staff would be beneficial. This would include, they said, informing staff about how to access research funding, encouraging non-researchers to suggest ideas, and feeding back findings to staff. An additional respondent suggested that a research champion should be introduced in each Directorate to highlight and promote research. In addition, five interviewees (26%) indicated that external communication to inform patients and the public about research should be improved.

##### Theme 4 – Reputation

When asked what the Oxford BRC brings to the OUH and to patient care, four interviewees (21%) volunteered that the Oxford BRC improves the reputation and profile of OUH. One of them felt that that the hospital is more likely to attract funding for improved physical infrastructure because of its high reputation, and that its high overall reputation is due in part to its high research reputation. Another explained that a high research reputation attracts more and better staff, as there are opportunities for networking, getting involved in the research programme and publishing. Reputation therefore also influences staff recruitment and retention (discussed further below), patient involvement, and access to high quality physical infrastructure.

Reputation *per se* is not mentioned as a distinct mechanism in the Hanney framework, although reputation is linked to the kinds of access to better infrastructure and human capital (staff) that are specified under the first of the Hanney mechanisms, ‘absorptive capacity’. We consider reputation to be distinct from absorptive capacity. Reputation is seen from a perspective outside the organisation, in this case OUH, whereas absorptive capacity is a characteristic of the people who work within the organisation and of how the organisation is set up.

##### Theme 5 – Staff recruitment and retention

Research (including that linked to the Oxford BRC) was identified as a key factor for attracting and retaining high quality clinical staff (medical and non-medical) to OUH. The impact on an organisation of being research-active is mentioned as part of the third item in the Hanney framework – ‘organisational mechanisms within healthcare systems’ – but we found it being given rather more prominence than that among the senior OUH clinicians we interviewed.

When discussing the positive effects of the Oxford BRC on OUH and patient care, nine interviewees (47%, including one of the divisional directors who was also an RT lead) mentioned that the Oxford BRC attracts good clinical staff to OUH. Two of these respondents added that, if the Oxford BRC were to be discontinued, staff members would be lost. This would not just be those staff members who are funded by the Oxford BRC, but also staff leaving to pursue research elsewhere. One interviewee was clear that “*research means a great learning environment*” that is attractive to high quality staff, and another commented that the Oxford BRC is a “*huge benefit to the* [OUH] – [the] *academic aspect draws in good people and its culture is appealing*”.

It was noted by two interviewees that research as a lure for high quality clinical staff is particularly important in Oxford as it is an expensive place to live: “*Oxford is expensive and it’s hard to recruit and retain staff, but doing research makes the job more interesting, gives variety, and it’s something other places don’t offer*”.

However, when discussing the possible challenges for OUH posed by the Oxford BRC, three interviewees expressed a concern that some of the best clinical staff may be attracted away from clinical posts and into research posts (see also Theme 2: formalisation of research roles). One interviewee explained that staff members sometimes move to research to get away from the more stressful environment of the NHS, and another noted that for doctors, nurses and other professional groups, moving from clinical work to supporting research usually means higher pay. In addition, two interviewees noted the loss of high quality clinical staff into research posts as a potential downside for patients of staff involvement in research.

##### Theme 6 – Patient benefits from staff involvement in research

The interviewees were explicitly asked about benefits and downsides for patients of staff being involved in research. Seventeen of 19 interviewees (89%) agreed that patients benefit from staff having direct involvement in research. The reasons given included that:

- Staff are better and more informed or interested (37%), which maps to the ‘absorptive capacity’ and ‘participatory research’ headings in the Hanney framework
- Patients receive more monitoring and staff contact/communication (37%), which overlaps with the Hanney framework’s ‘improvement in the processes of care related to conducting a specific trial’
- Patients get access to new treatments (21%), which is part of the ‘absorptive capacity’ and ‘participatory research’ categories in the Hanney framework
- Patients evidently appreciate that staff are interested in the condition they have presented with (21%), again part of the ‘absorptive capacity’ item in the Hanney list of mechanisms;
- It seems that patients are themselves better informed (16%), which can be seen as part of ‘participatory research’ in the Hanney framework
- Patients appear to enjoy contributing (11%), which is a spin-off from involving more patients in clinical trials and is not mentioned in the Hanney framework. (One interviewee commented that low drop-out rates from clinical trials at OUH (described as less than 5%) indicate high patient satisfaction. An illustrative example was given by another interviewee of a recent research project which involved comprehensive interviews with patients and families. Even the experience of undergoing the interview was thought likely to have had a positive psychological impact on the patients interviewed.

Of the two interviewees who indicated that there were no benefits to patients from staff being involved in research, one stated this was because the staff in their Directorate were providing a clinical support service and did not directly interact with patients. The other considered that the impact of research on clinical care may take time to become apparent, and there were no clinical changes that could be pointed to yet.

A variety of downsides to staff involvement in research were identified, although less frequently than upsides. Three interviewees (16%) stated that there were no downsides and another three offered no examples. However, five interviewees (26%; one of whom was one of the divisional directors who was also an RT lead) suggested that patients have to give up more of their time (although they may not mind and more contact may be seen as positive); three (16%) that research puts pressure on scarce space and/or equipment; and two (11%) that good nurses can be lost to research posts (see also Theme 2, formalisation of research roles, and Theme 5, staff recruitment and retention). Additional suggestions included unwanted side effects of novel treatments (e.g. chemotherapy), the risk of overburdening patients and that not all patients may be keen to take part.

##### Theme 7 – Access to infrastructure

Interviewees were asked whether research has meant a change in the infrastructure at OUH that is available for use in patient care. This item maps specifically to the first mechanism listed in the Hanney framework. Overall, 14 interviewees (74%; which includes both of the divisional directors who were also RT leads) said the infrastructure for patient care had been improved as a result of research activity including that by Oxford BRC, and five (26%) said it had not. Examples from the 14 yes-responders included whole genome sequencing, a digital pathology slide scanner, the clinical trials aseptic unit, laboratory facilities for flow cytometry, and a large resource centre including a library, which was built by the university. Interestingly, while one interviewee mentioned the AVIC as an example of additional infrastructure that has become available for patient care, another interviewee said that they were unable to access the AVIC due to the size of the usage fees for it.

Of the five interviewees who said no, one commented that research puts pressure on already scarce space, in particular on outpatient clinic slots when extra clinics or longer consultations are required by a research project. Another observed that, “*Generally, research is undertaken in dedicated research space and has little impact upon clinical infrastructure*”.

##### Theme 8 – Novel treatments and technologies

Seventeen interviewees (89%) agreed that OUH being research-active has helped OUH patients to access novel technologies, and 16 (84%) agreed that research has led to more patients going into clinical trials, although three of these stated that this does not automatically mean additional benefits for patients. The benefits of a novel treatment that are captured by the patients who participate in the clinical trial of that particular treatment are outside the scope of the Hanney framework. However, benefits of earlier access to novel technologies, which are felt by the rest of the hospital’s patients beyond those taking part in a specific clinical trial, are decidedly within the Hanney framework as a way in which patient care is improved in a research-active setting.

Specific examples of novel technologies which have become available at OUH due to research include (1) genetics patients getting much broader genetic testing than they would otherwise have had, leading to diagnoses that would not have been possible before, which in turn leads to more effective treatment; (2) fibroscan testing for liver disease; and (3) a new technique for cardio-resynchronisation therapy using a lead to pace the left ventricle of the heart, which is used to treat patients with heart failure. The new technique involves puncturing the septum (partition of the atria) and fitting the pacemaker that way.

One interviewee commented that, in addition to directly benefitting patients, earlier access to novel treatments and technologies increases clinicians’ awareness and familiarises them with these treatments. This increases their confidence in asking for and using novel medicines for their patients. It also means that other staff become used to using the novel treatments and technologies earlier than might be the case in other hospitals.

##### Theme 9 – Attitudes to research

Attitudes to research may, as with others of the themes, be seen as an (important) element of absorptive capacity. Three interviewees (16%) specifically noted that there has been a culture change at the OUH, as research has become more ingrained in day-to-day work, and two of these specifically linked the culture change to the Oxford BRC. Two further interviewees (11%) noted that staff often talk about studies and ideas, and a third commented: “*The people are motivated and interested and the team may be better. When there’s research, there’s more general interest in what is going on and how to do things*”. Four interviewees (21%) also noted that curiosity and personal interest are key factors for encouraging staff involvement in research. These are all aspects of improved ‘absorptive capacity’.

When asked whether staff have changed in their receptiveness to learning from research, nine of the 19 interviewees (47%) said yes (two of these nine were the divisional directors who were also RT leads), one of whom commented: “*The medical teams leading the research are making much bigger efforts to engage the staff on what they’re doing and there has been greater acceptance of research protocols being added to routine clinical treatment*”. Four interviewees (21%) said no, three of whom elaborated that staff in their part of the OUH have, in their view, always been receptive to research. The remaining six interviewees (32%) either did not answer, or provided mixed or ‘unsure’ responses.

The interviewees were also asked whether research has encouraged clinicians who are not involved in research to be more willing to use research findings. Eight interviewees (42%) answered yes, four (21%) said no (one of whom noted that this was because they recruit people who already have an interest in research), and the remaining seven (37%) either did not answer or provided mixed or ‘unsure’ responses. One interviewee who said no explained that “*Clinicians have always looked at research. However, the research is now more readily available*”.

##### Theme 10 – Collaboration

Interviewees were specifically asked who OUH staff interact with when conducting research, and whether there have been changes over time. Ten of the 19 interviewees (53%) mentioned the University of Oxford as a key collaborator (this included the two divisional directors who were also RT leads), four (21%) mentioned industry, two (11%) mentioned international collaborators, and one (5%) mentioned Oxford Brookes University. Six respondents (32%) indicated that there were collaborations with a variety of organisations.

Few respondents commented on whether or not the balance between these collaborations had changed over time. One commented that there had been no change over time in the collaboration with the University of Oxford (although it should be noted that this interviewee was much more familiar with the Biomedical Research Unit at the Nuffield Orthopaedic Centre than with the Oxford BRC), whereas another commented that during their time as a consultant there had been a “*steady improvement in NHS/university relations towards more collaboration*”.

Collaboration is likely to be closely linked to many of the other themes that emerged from the interviews with senior OUH clinicians. Adams [14] describes the benefits of collaborative working as “*It provides access to resources, including funding, facilities and ideas*”. Increases in collaboration are likely to influence the amount of research activity (Theme 1), communication and awareness of research (Theme 3), reputation (Theme 4) and therefore staff retention (Theme 5) and patient benefits from staff involvement (Theme 6), access to infrastructure (Theme 7), access to novel treatments (Theme 8), and attitudes to research (Theme 9).

#### Individual projects

The interviewees were asked whether they were aware of any specific Oxford BRC-related projects. Twelve of them identified one or more specific projects. In all, 24 different projects were mentioned unprompted by at least one of the clinicians interviewed. We have confirmed 16 of these as being Oxford BRC-related projects (12 of them were in the list of projects suggested by RT/WG leaders; five of them were mentioned by the divisional directors who were also RT/WG leaders). One project (SEND) was mentioned by two different interviewees, and was the only project to receive more than a single unprompted mention. The senior clinicians identified 10 of the 16 projects as having had an impact on patient care at OUH, but in most cases we did not obtain further details about the type of impact.

When prompted about a set of specific Oxford BRC-related projects or clinical service initiatives (selected as most likely relevant to the interviewee from the list of 44 compiled from the RT/WG interviews), interviewees considered that 11 had had an impact or potential impact on patient care at OUH, making 21 (48%) impactful BRC projects in total (Table 2). All interviewees said they were aware of at least one of the projects/initiatives suggested to them, even if they could not provide further details about it. Comments made by the interviewees indicated that they were aware of a range of research projects taking place in their Directorate or Division and involving University collaboration, but they often did not know which were linked to Oxford BRC. Unsurprisingly, interviewees were most familiar with projects they had some direct involvement with.

**Table 2:** The Oxford Biomedical Research Centre (BRC) projects that Research theme/Working group leads indicated had potential impacts on patient care at Oxford University Hospitals NHS Foundation Trust (OUH), with an indication of whether that impact was perceived by senior clinicians at OUH

BRC Research theme/Working group	Oxford BRC projects with potential impact on patient care	Potential impact on patient care at OUH	OUH senior clinician mentioned impact unprompted?^a^	OUH senior clinician confirmed impact after prompting?^a^
Biomedical Informatics and Technology	Gestational diabetes smartphone application	Processes and/or service organisation	YES	n.a.
	New alerting score based on patients’ vital signs (CALMS-3 Study – Computer Alerting Monitoring System)	Processes and/or service organisation	NO	NO
	Maintenance of database of samples and consent management for Oxford Biobank	Processes and/or service organisation	NO	YES
	System for Electronic Notes Documentation (SEND) project (electronic track and trigger and patient alerting)	Processes and/or service organisation	YES	n.a.
Blood	Training and hiring staff and improving infrastructure for clinical programme to do stem cell transplants and run more clinical trials in this area	Treatments	NO	NO
Cancer	Multi-gene testing service with rapid (1 week) turnaround	Diagnostics, testing, screening	NO	YES
	BRC-funded clinical research posts specialising in sarcoma, gynaecological cancers and melanoma	Staffing for specialist services	NO	NO
Cardiovascular	MRI diagnostic tools for patients with intermittent angina	Diagnostics, testing, screening	NO	YES
Clinical Informatics	Upgrade for cancer informatics in OUH (enabled by Genomic Medicine Centre (GMC) designation)	Diagnostics, testing, screening	NO	NO
	Molecular diagnostics and genome sequencing services (enabled by GMC designation)	Diagnostics, testing, screening	NO	NO
	Development of integrated logical record for each cancer patient (in progress)	Processes and/or service organisation	NO	YES
	True colours technology for mental health patients across Oxfordshire to aid self-management	Processes and/or service organisation	NO	YES
	Streamlining management of notes/records from multidisciplinary cancer meetings (in progress)	Processes and/or service organisation	NO	YES
	Clinical decision support dialogue to make blood orders more efficient	Processes and/or service organisation	NO	NO
Dementia and Cerebrovascular Disease	New screening process for acute confusion in medical admissions	Diagnostics, testing, screening	NO	YES
	Fast track carotid surgery	Treatments	NO	NO
	Telemetric home blood pressure monitoring	Processes and/or service organisation	NO	NO
	The first transient ischaemic attack (TIA) and minor stroke clinic in the United Kingdom	Processes and/or service organisation	YES	n.a.
Diabetes	Diagnostic programme to identify patients with a genetic basis for their diabetes	Diagnostics, testing, screening	NO	NO
	Pancreatic islet extraction unit	Treatments	NO	NO
	Pancreatic transplants	Treatments	NO	NO
Functional Neuroscience and Imaging	Imaging protocols (epilepsy)	Diagnostics, testing, screening	NO	NO
	Functional neurosurgery team (part of the BRC) provides a clinical service, including pain deep brain stimulation	Treatments	NO	NO
	Specialist clinic in epilepsy, Parkinson’s disease, motor neurone disease, supported by BRC-funded research fellows	Staffing for specialist services	NO	NO
Genomic Medicine	Targeted next generation sequencing	Diagnostics, testing, screening	NO	NO
	Whole exome or whole genome sequencing	Diagnostics, testing, screening	NO	NO
Immunology and Inflammation	Non-invasive tests for liver disease	Diagnostics, testing, screening	YES	n.a.
	Patient stratification for biologic therapies	Diagnostics, testing, screening	NO	YES
	Tests to diagnose encephalitis (brain inflammation)	Diagnostics, testing, screening	NO	NO
	Trials on treatment of inflammation in eczema	Treatments	NO	NO
Infection	Work linking care to outcomes with big data approaches	Processes and/or service organisation	NO	NO
	Genomic testing to support infection monitoring (provided informally)	Processes and/or service organisation	NO	NO
Molecular Diagnostics	Oxford designated as GMC for Genomics England’s 100,000 Genomes Project	Diagnostics, testing, screening	YES	n.a.
	Multi-gene testing for cancer patients	Diagnostics, testing, screening	NO	YES
	Diagnostic tests for children with rare blood conditions	Diagnostics, testing, screening	NO	NO
	Response prediction in leukaemia	Diagnostics, testing, screening	NO	YES
Prevention and Population Care	The first TIA and minor stroke clinic in the United Kingdom	Processes and/or service organisation	YES	n.a.
Surgical Innovation and Evaluation	Chemotherapy (device targeted therapy): liposomes developed to carry the treatment to the organ of interest	Treatments	NO	NO
	Image-guided surgery using fluorescents	Treatments	YES	n.a.
	Organ transplantation/reconditioning	Treatments	NO	NO
	Restoring vision through an electronic implant	Treatments	YES	n.a.
	Restoring vision through gene therapy	Treatments	YES	n.a.
Training and Education	No projects identified			
Translational Physiology	No projects identified	Diagnostics, testing, screening	YES	n.a.
Vaccines	Respiratory syncytial virus, trialling a new vaccine^b^	Treatments (national)	YES	n.a.
	Meningitis, looking at trials of schedules for baby vaccines, booster doses, meningitis B vaccine trials^b^	Processes and/or service organisation (national)	YES	n.a.
Multiple Research themes/Working groups	Better infrastructure for clinical trials and other studies (e.g. better pharmacovigilance, biobanking)	Processes and/or service organisation	NO	YES

NO, Either none of the clinicians interviewed recognised the project as described, or if it was recognised it was not confirmed as having an impact on patient care; YES, At least one clinician interviewed recognised the project and confirmed impacts from it; n.a., Not applicable

^a^The absence of a YES from both of the last two columns of the table should not be interpreted as evidence of an absence of clinical impact. The approach used to query impacts had limitations. Each interviewee was prompted with a list of 3–6 projects selected by the interviewers before the interview on the basis of the interviewee’s clinical area, so lack of recognition of projects could result from (1) unsuitable choice of prompts by the interviewers, (2) projects not being used as prompts, or (3) the projects may not have been described in terms familiar to the interviewees

^b^Projects were not among those prompted, because their impact is national rather than specific to OUH, but they were both mentioned by clinicians unprompted

In addition to the projects in the table that were mentioned unprompted, four projects were mentioned unprompted and confirmed to be Oxford BRC projects: digital monitoring of neonates in an intensive care unit, research on radiotherapy for rectal cancer, Tardox project to deliver treatment to specific tumour sites in liver, and metabolic imaging of brain tumours. A further eight projects were mentioned unprompted by the senior clinicians when asked about Oxford BRC projects that might have impacted patient care, but these had not been mentioned by the Oxford BRC RT/WG leads and were not identifiable from Oxford BRC documentation as being projects they had contributed significantly to

#### Cross-analysis of results from RT/WG interviews and senior clinician interviews

Comparing the comments made by both sets of interviewees – RT/WG leads and senior clinicians – allowed us to assess the themes from two different perspectives and obtain a more complete picture of the Oxford BRC’s impacts on the OUH. RT/WG interviewees spoke in depth about the research they are involved in and often identified a range of positive impacts of that work, including academic impacts. The senior clinicians, who are responsible for managing and delivering clinical services, had a strong awareness of the practical implications of research activity.

Unsurprisingly, the RT/WG leads expressed predominantly positive views about research in general. The senior clinician interviewees presented a more mixed picture, although still more often positive rather than negative about research. Differences in views that arise tend to reflect the interviewees’ differing roles and responsibilities. A summary of the overall positive and negative findings under each theme, with an indication of which groups of interviewees made the relevant comments, is presented in Table 3.

**Table 3:** Summary of analysis across Oxford Biomedical Research Centre (BRC) research theme/working group (RT/WG) leads and Oxford University Hospitals NHS Foundation Trust (OUH) senior clinician (SC) interviews, organised by theme

Theme	Positive changes mentioned (interviewee type)	Challenges and risks mentioned (interviewee type)
1. Research activity	Research activity has increased over time (SC and RT/WG) The Oxford BRC plays an important role in enabling research to happen and helps attract additional research funding (RT/WG)	For some, it is unclear how decisions are made about which clinical areas receive Oxford BRC support (SC) The types and topics of research taking place may not fully align with OUH clinical needs (SC)
2. Formalisation of research roles	There has been an increase in the number of medical and non-medical clinical research staff (SC and RT/WG) More clinical staff have time protected for research, which better enables them to engage in research (SC and RT/WG)	The fixed-term, part-time nature of Oxford BRC research posts can create tension for OUH staff organisation and planning (SC)
3. Communication and awareness of research	Staff awareness of ongoing research and associated opportunities has increased in some clinical areas (SC) Oxford BRC has increased the profile of patient engagement in research through multiple initiatives (RT/WG)	Clinical staff should be better informed about research taking place, opportunities to get involved and findings (SC) External communications could be improved (SC)
4. Reputation	Oxford BRC improves the reputation and profile of OUH (SC and RT/WG)	None mentioned
5. Staff recruitment and retention	Staff are attracted to the OUH because they believe the Oxford BRC and links to the University of Oxford will create opportunities for research and career development (SC and RT/WG) Research opportunities may encourage staff not to leave the OUH (SC)	High quality staff (especially non-medical staff) move into research posts and out of clinical work (SC mainly, but also RT/WG)
6. Patient benefits from staff involvement in research	Staff are better informed about developments in treatments (SC) Staff reflect more on clinical decisions and how to deliver care (SC and RT/WG) Patients interact with staff more; they may receive better care and feel better cared for (SC) Patients gain access to new treatments (SC) Patients report that they enjoy being involved in research and feel they are contributing to the public good (SC and RT/WG)	Patients may feel inconvenienced or overburdened, particularly if study design and communication to patients are poor (SC)
7. Access to infrastructure	Additional, improved or lower-priced infrastructure has become available in some areas because of research (SC and RT/WG)	In other areas, there may be opportunities to share infrastructure which are not being realised (SC) Research activity can put additional pressure on clinical infrastructure (SC)
8. Novel treatments and technologies	Many patients have had access (or earlier access) to novel treatments and technologies because of research (SC and RT/WG)	None mentioned
9. Attitudes to research	In some areas staff have become more interested, motivated and willing to use research findings (SC)^a^	Some perceive a split between the University and NHS; some staff never engage with research and may feel ‘outside’ of research (SC and RT/WG)
10. Collaboration	Oxford BRC brought more collaboration between Oxford University and the OUH; it made research more clinically relevant and raised the profile of research in the OUH (RT/WG mainly, but also SC)	None mentioned

^a^In some areas, interest and willingness to use findings has been high for a long time, or has increased through a wider shift towards evidence-based medicine

Interviewees from both groups agreed that there has been an increase in research activity (Theme 1) in recent years, although some senior clinician interviewees questioned how clinical areas were chosen to receive support from the Oxford BRC. RT/WG interviewees indicated that the Oxford BRC has helped to drive the increase in research by providing research infrastructure and helping to attract research funding. They explained that the Oxford BRC had been able to bring about this change in part because it had enhanced the reputation of the OUH (Theme 4), a view that was strongly supported by senior clinician interviewees. The presence of the Oxford BRC was widely recognised as a badge of high quality.

RT/WG interviewees thought that the Oxford BRC had brought about more collaboration between the OUH and the University of Oxford; however, this observation was made by relatively few senior clinician interviewees. Some senior clinician interviewees did report that staff had become more receptive to and interested in research, a view consistent with RT/WG interviewees’ comments that research bringing about positive changes in attitudes to research (Theme 9). A minority of interviewees from both groups felt that there was still a clear divide between the two institutions, and between researchers and non-researchers.

The shift towards formal research roles for clinical staff was widely discussed by both sets of interviewees (Themes 2 and 5). RT/WG interviewees emphasised the positive benefits of protected research time, which enables clinical staff to engage more in research, and of support from research nurses. However, senior clinician interviewees, many of whom were involved in job planning as part of their clinical roles, observed that it can be challenging to plan around these posts in the NHS environment. Multiple senior clinician interviewees also voiced a concern that nurses and other non-medical staff are being lured away from clinical work to the better pay and conditions available in research posts. Still, both sets of interviewees agreed that the Oxford BRC, and the Oxford environment more generally, help to attract high-quality staff.

Comments made by senior clinician interviewees indicated that research opportunities are not always being effectively communicated to OUH staff (Theme 3). Although some said improvements had been made in this area, it was still often cited as an area for improvement. There was seen to be a need for better communication about what research is taking place, as well as an increase in opportunities to get involved. When prompted about specific Oxford BRC projects and initiatives, senior clinician interviewees occasionally expressed surprise that projects were considered ‘Oxford BRC projects’, highlighting some ambiguity about what it means for a project to be an Oxford BRC project and, potentially, a need for better communication about Oxford BRC activities. RT/WG interviewees, on the other hand, discussed how a range of opportunities (e.g. for training) were available and how the Oxford BRC was supporting various initiatives to encourage researchers’ engagement with patients and the public.

A range of benefits for patients, including access to novel treatments and technologies (Theme 8), and infrastructure (Theme 7), were identified by both groups of interviewees. However, some senior clinician interviewees commented that research can put extra pressure on clinical infrastructure and indicated that not all major equipment is made available for wider use. Senior clinician interviewees in particular discussed other patient benefits from staff involvement in research (Theme 6), including patients having more contact with staff and staff becoming better informed about clinical research developments. Many of the senior clinicians we interviewed felt that patients enjoy being involved in research, but some acknowledged a risk that patients could be overburdened by it. Three RT/WG interviewees described how clinicians who do research are exposed to different approaches and reflect more about how to approach their work.

As mentioned previously, comments made by the two divisional directors who were also RT/WG leads were not markedly different from those of the other RT/WG leads. During the senior clinician interviews, these two interviewees highlighted many important benefits of the Oxford BRC. For example, both felt that clinical staff had become more interested in research over time, both felt that there had been benefits to patients of being included in more trials where they get closer monitoring, and both felt that staff had increased in their receptiveness to learning from research. When asked what would happen if the Oxford BRC was discontinued, one commented that it would be “*tragic, unthinkable, a major setback*”. Perhaps unsurprisingly, these interviewees felt that more funding and more research would further benefit OUH.

#### Fit with the Hanney framework

In our discussion of the themes emerging from the interviews with senior clinicians at OUH, we have noted the extent to which they relate to the mechanisms listed in the Hanney framework as possible ways in which research activity at a healthcare provider might affect the care received by its patients. In the following paragraphs, we go through each of the Hanney mechanisms in turn, summarising the extent to which the evidence from the OUH senior clinician interviews corresponds to them.

##### Absorptive capacity

Interviewees did report improvements in infrastructure (Theme 7), human capital (Themes 2 and 5) and attitudes towards research (Theme 9), which have an impact outside of research activities, i.e. on patient care. Changes in human capital were mainly linked to improvements in knowledge, motivation, awareness and attitudes, rather than additional personnel. The Hanney framework suggests that additional personnel may be brought in to do research and then remain afterwards to the benefit of patient care, but this does not appear yet to have been the case at OUH. However, several interviewees commented on the possible recruitment advantages of the enhanced reputation that research activity, including Oxford BRC, brings to OUH.

##### Improvements in the processes of care related to conducting a specific trial

Twelve of 19 interviewees (63%) stated that research had beneficially affected the way that care is delivered. Interviewees also reported that more patients are being enrolled in clinical trials, and that research has helped OUH patients to access novel technologies (Theme 8).

##### Organisational mechanisms within healthcare systems

Research does appear to have had an influence at the organisational level. Some interviewees mentioned a culture change (Theme 9). The organisation of personnel has also been altered to some extent (through the formalisation of research roles and protecting some people’s time for research; Theme 2), thereby making the organisation a more attractive place to work (Theme 6) and enhancing its reputation (Theme 4). The presence of research has also encouraged non-research clinicians to be more willing to use research findings in some, though not all, areas of the OUH (Theme 9).

However, challenges remain here, as some of the most experienced clinical staff are then attracted out of clinical posts into research roles, which then may have a negative impact on human capital and absorptive capacity for patient care.

##### Collaborative approaches between organisations, teams and individuals

As discussed earlier, collaborative approaches between the University of Oxford and OUH were important; such collaboration is a requirement of BRC-funded research. Alongside this, and probably at least partly due to collaboration, eight of 19 interviewees (42%) felt that the relevance of research has increased, and only two (11%) felt that the relevance had not increased. The remaining nine interviewees (47%) provided mixed or unclear responses.

##### Action and participatory research

No interviewee referred explicitly to ‘action research’ or ‘participatory research’ as particular ways of conducting research. These are approaches which involve the participation of research users in planning, designing and participating in research [10]. However, increased staff and patient participation in research was described on numerous occasions during the interviews. Research activity by clinical staff has increased (Theme 1, research activity) and patients were seen as benefiting from that (Theme 6). Further, as mentioned in the discussion of Theme 9, attitudes to research, 42% of respondents felt that research had encouraged clinicians to be more willing to use research findings.

##### Summary with respect to the Hanney framework

All of the five types of mechanisms suggested in the Hanney framework by which research-active settings may provide better patient care were evident, at least to some degree, in the senior clinicians’ responses. That is particularly true of enhancement of absorptive capacity (both infrastructure and human capital), improvements in processes of care related to conducting specific clinical trials, and organisational mechanisms within healthcare systems. Although better collaboration between OUH and University of Oxford staff was mentioned, impacts being achieved via collaborative approaches between them and third party organisations and networks were not referred to.

The first set of mechanisms categorised by Hanney et al. [10] as ‘absorptive capacity’ includes both infrastructure and human capital. The frequency with which each of these kinds of impacts was mentioned suggests it may be helpful to separate them. Furthermore, we identified impacts from research activity which, while clearly connected with infrastructure and human capital, had their impacts on patient care via a more direct route than by increasing the organisation’s absorptive capacity. Thus, research activity led to the provision of physical infrastructure or human capital that was then also available for patient care activities (as distinct from research activities) part of the time. Where this was noted, it represents a benefit to patient care that appears to us to be quite distinct from the improved ability of OUH and its staff to also take more advantage of research conducted elsewhere.

Finally, we consider that the impact of research activity on OUH’s reputation, which in turn can lead to patient care benefits, is worthy of explicit mention as an additional item in the list of mechanisms.

## Discussion

The care provided by healthcare organisations whose staff are more active in research may be affected by that research activity, and these effects may occur indirectly. That is, they may arise independently of any direct impacts from implementing the outputs of successful research [10, 12, 15]. Hanney et al. [10] provided a five-point list of mechanisms through which healthcare might be improved in research-active settings, which we have labelled ‘the Hanney framework’. We have undertaken a qualitative analysis of the extent to which a major NHS hospital foundation trust, OUH, bears out that view, with particular reference to the research activity that is related to the Oxford BRC.

The time required for different types of impacts to occur varies significantly, and this influenced our study. Given the long and variable time lags between even translational research – which is the focus of BRCs – and consequent benefits (if any) directly stemming from it, it is too early to detect an impact at OUH directly from the outputs of research projects related to the Oxford BRC [16]. The potential indirect impacts can be expected to occur with rather shorter lags, however. Some of the mechanisms identified by Hanney et al. [10] could have an almost immediate impact, e.g. benefits to routine processes spilling over from increased numbers of clinical trials, though others would only have an impact over time, e.g. greater receptiveness towards research findings generally.

The leaders of the RTs and WGs within the Oxford BRC identified numerous ways in which they expected Oxford BRC-related research activity might benefit the care provided by OUH to its patients. Such expectations are unsurprising. We therefore tested whether the senior clinicians responsible for patient services in OUH also detected any such impacts. We simultaneously tested the applicability of the Hanney framework as a way of structuring the analysis.

The Oxford BRC is part of the overall medical and healthcare research portfolio conducted in Oxford. Our questioning of senior OUH clinicians therefore focused first on whether they were detecting impacts from research activity in general. We did not ask them to apportion responsibility for any impacts between different parts of the research portfolio, but we did then test the extent to which they were aware of how the Oxford BRC specifically was contributing to the overall research endeavour visible in OUH. Overall, we found that the majority of OUH senior clinicians consider there to be noticeable impacts on patient care as a result of the research activity going on. The impacts they identified were consistent with those expected by the RT/WG leads for Oxford BRC. The impacts identified by the senior OUH clinicians are on balance beneficial, although some negative consequences were identified alongside the larger number of positive ones. Many of the senior clinicians interviewed cited particular Oxford BRC-related research that was impacting the clinical services for which they were responsible.

The period of operation of the Oxford BRC, since April 2007, has seen a perceived increase in the quantity of research activity at OUH, a greater quantity of explicit research collaboration between the University of Oxford and the OUH (which is a condition of NIHR BRC funding), and greater formalisation of who undertakes research. We note that an even distribution of research is neither expected nor necessarily desirable, as there will be higher need for research and/or higher potential returns to research in some areas compared to others. Findings from the senior clinician interviews suggested that the increase in research activity at OUH has not been uniform across all clinical Directorates/Divisions. It was outside the scope of the present study to assess how research was distributed by the BRC and how decisions about research priorities were made, but this is an area that is worth future consideration and research.

The increased formalisation of research roles is of particular interest and appeared to create tension in terms of staff organisation and planning. Its positive consequence is that OUH staff are reporting clearer protection of work time for research, giving them the ‘head space’ to think about and pursue research ideas while continuing to provide patient care with the rest of their time. Research opportunities have greater profile among hospital staff. However, these positive outcomes – of a more formal distinction between those with paid research time and those without – have the corollary that some staff feel excluded from research. Interviewees did not comment on whether this results in a net negative or a net positive impact on OUH patient care overall.

Generally, it seems that Oxford BRC and other research activities at the OUH have had a mixed effect on staff retention and recruitment for patient care. These factors attract people to work at OUH, but can then also take them out of clinical work into research posts. It was suggested that the attractions of, and greater opportunities for, a research post may lead to the loss of some high quality nursing staff from patient-facing work. The net impact of greater research activity making OUH a more attractive place to work on the one hand, but taking some experienced staff away from NHS work on the other, is unclear and might be a worthwhile focus of future more detailed research.

The reputational benefit of being seen as a research active hospital, and the contribution of the Oxford BRC to enhancing that activity and that reputation, came up several times. Reputation per se was not included by Hanney et al. [10] in their framework. It can be seen as underlying and reinforcing mechanisms included in the framework: higher reputation institutions may find it easier to recruit and retain higher quality staff, other things being equal, and may find it easier to attract further research funds and associated infrastructure. However, improved reputation as a result of research activity may also spill over into greater confidence among the population served by the hospital that they will receive high quality care when they are patients there. There may be a reassurance benefit to patients and the local population in general, in other words, which makes reputation another medium by which research activity may translate into patient and population benefits. Reassurance value is a recognised category of benefit, but one which is hard to measure and value [17].

The majority of the senior clinicians we interviewed saw research, including Oxford BRC-related research, as leading to improved access to physical infrastructure. A small number noted a downside, however, in the form of increased research activity, putting greater pressure on outpatient clinic capacity and throughput. Although some provision for capital funding was made in the NIHR’s initial BRC awards in 2007, the rules of further quinquennial rounds of BRC funding in 2012 (and 2017) mean that it cannot be used for capital expenses such as major equipment or buildings, although it can contribute to the cost of operating the capital equipment/buildings obtained from other sources of finance. Furthermore, BRC funding can help to fund other types of infrastructure such as (non-clinical) support staff.

In general, receptiveness to research among OUH staff appears to have increased over time, although three of the senior clinicians interviewed considered that, in their areas, research receptiveness had always been good. Awareness among OUH staff of Oxford BRC-related research projects extended to a reasonably large number of the projects in the current Oxford BRC portfolio (we note that interviewees’ ability to recognise projects may have been affected by our choices about which projects to prompt them on and the descriptions we provided).

Issues of communication and awareness were, however, a theme across the senior clinician interviews. Interviewees stressed the importance of raising awareness and providing opportunities for staff to participate in research, and five senior clinician interviewees said that increasing awareness among staff of research being carried out would enable OUH to benefit more from that research. It seems that some Divisions and Directorates at OUH have already seen improvements in this area, but others less so. There is room for further improvement in communication and awareness of research across OUH. Senior clinicians interviewed suggested that it would be helpful to inform staff about how to access research funding, encourage non-researchers to suggest ideas, ensure findings are fed back to staff, appoint a research champion in each directorate to promote research, and run seminars and display research posters. Five senior clinician interviewees also indicated that communication to patients and the public about research should be improved.

In addition, 17 out of 19 senior clinicians considered that patients benefit from OUH staff being involved in research. These two facts combined imply that Oxford BRC, by increasing research activity, and specifically collaborative research activity, can be viewed as making a significant contribution to improvements in patient care. A small number of specific examples of novel technologies becoming available due to research at OUH were also mentioned (improved genetic testing/diagnosis, fibroscan test for liver disease, improved cardio-resynchronisation therapy).

There was also some confirmation that the Oxford BRC, by the nature of its requirement for collaborative working between the University of Oxford and OUH, had led to an improvement in relations between the two organisations. Collaboration between research and patient care has a higher profile now than previously and offers the prospect of better, more relevant and more readily adopted (locally at least) research, and with no apparent negative consequences for patient care.

In conducting the study we found the framework proposed by Hanney et al. [10] to be useful and relevant for structuring the interview protocol and analysing the responses we obtained. In addition, we would highlight that infrastructure and human capital created for research can directly benefit patient care as well as doing so by improving a healthcare provider’s absorptive capacity. Furthermore, the number of references that were made to the positive impact of research, and of Oxford BRC funding status for some of that research in particular, on the OUH’s reputation among staff and patients, suggest to us that ‘reputation’ is worthy of inclusion in an extended taxonomy of mechanisms by which research-active settings produce greater patient benefits. The reassurance that they are receiving high quality care, or will receive that when they need it, contributes to the wellbeing of the local population and of patients. Staff morale, and hence performance, are both likely to be enhanced by a higher reputation for the organisation they work in.

### Limitations

Our qualitative study suffers from inevitable limitations. It does not enable the quantitative estimation of the scale of any net (dis-)benefits on healthcare at OUH of the Oxford BRC’s research activity. Disentangling the quantitative impact of the various mechanisms through which research activity could have indirect impacts from the many confounding factors would be problematic; our approach qualitatively attempts such disentangling.

Inevitably, we are exposed to the possibility that interviewees’ responses are affected by incomplete or biased recall. What was said in the interviews should not be taken to represent a full statement of all BRC impacts. We interviewed the RT/WG leads of Oxford BRC, with the expectation that they might, taken together, tend to an optimistic view of the impact of their research on clinical care at OUH. The purpose of the RT/WG leads was to enable full preparation for the subsequent interviews at OUH, but not to be the source of definitive evidence of impact; for that we rely on the evidence of NHS staff at OUH. As described earlier, we found much consistency between what OUH senior clinicians told us and what the Oxford BRC RT/WG leads expected; but some of the clinicians referred to negative impacts, which RT/WG leads might not have been aware of and had not mentioned.

We selected the clinical heads of Directorates and Divisions as our interview targets at OUH in order to balance sufficient direct knowledge of the patient care services being delivered, against being able to cover the full width of NHS services provided there, within the constraints of the project budget and timescale, and being able to tap the knowledge of people with several, or more, years of experience of working at OUH. Interviewees at OUH knew we were approaching them for a study funded by a grant from the Oxford BRC, and we structured the interview protocol in order to prompt consideration of all mechanisms by which research might impact, positively or negatively, on patient care. Thus, the interviewees would have been sensitised to issues about any possible links between research activity, specifically research activity related to the Oxford BRC, and patient care. However, we were careful to avoid leading questions. Further, during the interviews we did not remind interviewees of any Oxford BRC research until after we had obtained their views on any impacts from research.

It may be that, on average, clinicians who are working in a more research-active setting are likely to be more favourably disposed to seeing benefits from working in a research active setting, than would, on average, clinicians who are working in a less research active setting. Qualitative analysis in a research-active setting, such as OUH, cannot avoid that issue. Nevertheless, qualitative analysis in a relatively research-inactive setting could not be expected to identify the impacts of research. Future study might therefore be directed to identifying and then collecting, at the level of individual patient care services where our qualitative analysis suggests there has been an impact, quantitative indicators of impacts on specific patient care services. Nevertheless, the clinicians we interviewed proved ready to describe negative impacts as well as positive, so any potential degree of bias appears likely to have been subtle rather than crude.

## Conclusions

We identified a range of impacts from research on the provision of patient care at OUH. Indirect impacts, which are not generally subject to the long time-lags associated with direct impacts, were particularly notable. Oxford BRC-related research contributed to those impacts and to the growth in research activity that has been experienced in general at OUH over recent years:

- Patients have had earlier access to novel treatments and technologies as a result of research activity locally
- Research activity has become more formalised in recent years, bringing advantages from funded research time for OUH’s NHS staff, but tensions with those staff who are not so funded
- NHS staff awareness of research appears to be increasing, though there is still work to be done to improve communication about research that is going on, opportunities to get involved, and the findings that emerge
- The Oxford BRC adds positively to the reputation of OUH
- Staff recruitment and retention may be improved in aggregate, though there are local issues with experienced staff being drawn into research and away from patient care
- Patient involvement has increased over time, associated with a major increase in clinical trial activity at OUH
- Additional physical infrastructure has become available in some areas, although this is constrained by Oxford BRC funds not being allowed to be used for capital equipment purchases; against that there were comments to the effect that the demands of extra research activity involving patients can increase pressure on NHS care facilities such as space for outpatient clinics
- In some areas, staff have become more interested in and receptive to research findings, although in some areas such interest and receptiveness have been high for many years

Impacts from research activity on the effectiveness and efficiency of patient care at local acute hospitals, as perceived by senior clinicians responsible for delivering that care, were numerous and usually positive, although occasionally negative. The senior clinicians made practical suggestions for improving that impact, for example, by better communication, and hence awareness, of research.

The taxonomy of mechanisms described by Hanney et al. [10] provided a helpful basis for conducting the analysis and categorising the findings. All five of the categories of benefit in the Hanney framework were evident in our particular study. Additionally, the impact of research in general and of the Oxford BRC in particular on the reputation of the healthcare provider organisation (OUH) seems worthy of distinct consideration in the framework of mechanisms by which research activity at a provider benefits (or disbenefits) patients.

Finally, a number of directions for further research to better understand the impact of research activity on patient care suggest themselves. Not least, it would be potentially highly informative to undertake research with a wider range and greater depth of staff, including non-medical staff, who are responsible for providing care to patients. This could be most practically done by focusing on a small sample of clinical services. As well as going into greater depth, it would be informative to research the extent of any spillovers from OUH to other parts of the local health economy, particularly primary care and other community based care. Of equal interest could be prospective analysis, monitoring research in real time and monitoring care provision at the same time in the areas where that research activity is expected to impact most. It would also be helpful to explore how research is prioritised and distributed by the BRC across different areas and, potentially, improve transparency and communications around this in the OUH. Comparison between the experience of patient care at OUH, at a comparable research-active Hospital Trust which is not part of an NIHR BRC, and at a comparable (in terms of service mix and scale) Hospital Trust that is significantly less research-active, would also be valuable.

## Additional files

Additional file 1: Research theme and working group leaders: interview protocol. (DOC 33 kb)
Additional file 2: Protocol for interviews with clinical staff at Oxford University Hospitals NHS Foundation Trust. (DOC 34 kb)
