# Supplementary material for: Does a biomedical research centre affect patient care in local hospitals?
Source: Health Res Policy Syst. 2017 Jan 21;15:2. doi: 10.1186/s12961-016-0163-7 (PMC5251230; doi:10.1186/s12961-016-0163-7)
Supplement: Additional file 1: — Research theme and working group leaders: interview protocol. (DOC 33 kb) [file 12961_2016_163_MOESM1_ESM.doc]

**Additional file 1:**

**Research Theme and Working Group leaders: Interview protocol**

**Background information:**

- **Project goals:** We are carrying out this evaluation to better understand what the impacts of the BRC have been for the OUH specifically – to inform the bid for BRC funding renewal and also to see if there are things that could be changed or done better, or things that are working well and should be recognised and continued or supported more.
- **Project overview:** Our evaluation has three parts - two sets of interviews and a bibliometric analysis:
  - First set of interviews: With RT/WG leaders to get a sense of what you expect the impacts may have been at OUH (and in health care more widely) related to work in your theme (or working group).
  - Second set of interviews: With senior clinical staff in the hospital to get their perspective on the impacts of the BRC- their views in general and also related to what the research leaders tell us. We will speak to people who are likely to have had some contact with research, but the interviewees will not necessarily need to know very much about what the BRC is – the important thing will be their experiences ‘on the ground’.
  - Bibliometric analysis: to gather publications data about the impact the BRC has had on the quantity, type and quality of research and collaborations between the University of Oxford and OUH, comparing with locations that do not have a BRC.

Note: The University of Oxford Health Economics Research Centre (HERC) with Prof. Alastair Gray is doing a study on specific impact case studies. Our work has a broader focus – looking at wider impacts (including direct effects of BRC research and more indirect changes in the awareness of research and attitudes towards research in the clinical setting, which affect the quality and/or cost of patient care provided by OUH).

**Confidentiality and recordings:**

- We will take notes during the interview, and (if you agree) make a recording for our own internal use - to refer back to check details.
- The notes and recordings will only be available to our research team.
- Recordings will be deleted at the end of the project.
- In the outputs from this work – the next set of interviews, an internal report for the BRC and possibly an academic publication – no comments will be directly attributed, or otherwise identified, to you.
- We will share our notes (not full transcripts but key messages) by email within two weeks of the interview for you to review and check accuracy.

**Interview questions will cover:**

- **Specific projects** and activities in your part of the Oxford BRC that you think may have been important in terms of having a **direct impact** on the OUH and/or its patients – what these impacts were and how they came about. Direct impacts are changes to healthcare, like a new diagnostic tool or treatment or a reorganisation of process becoming available that enables better care or the same care but at reduced cost or patient burden.
- More **indirect impacts** related to the BRC – like provision of equipment or training that benefits more than the research it was obtained for, and like changes in attitudes to participation in, and implementation of, research. These impacts could be supported by evidence of increased collaboration with Oxford University or other universities, or greater interest in implement research findings from whatever sources. (These could have come about through specific projects or through more general interactions that have happened.)
- If there are examples of a **lack of impact or of negative impacts**, these are also worth talking and thinking about. We are not just interested in the success stories but also in finding out what might not be working so well, and maybe ways that things could be done better.
- We are interested in impacts beyond the OUH (e.g. other healthcare settings), but for the current project we have time and budget constraints, so we have to limit the scope and are focusing on OUH impacts and economic/industry impacts.

**Interview questions**

**Introduction**

1. Could you describe yourself and your role in the BRC?
2. How long have you been in this role (at BRC and as a researcher in Oxford)?
3. How is your Research Theme/Working Group structured and how does it work, generally? (e.g. Who is directly involved from the academic and clinical sides? Which clinical units at the OUH are involved?)

**Direct impacts**

1. Could you tell me what are examples of specific projects that have been part of your theme that have had impacts on the OUH – improving patients’ health and/or experience of care, and/or cost savings for OUH?
2. Can you describe the impact and how it came about?
3. Why did you do this work?
4. How did the BRC contribute to achieving this impact?
5. Was this research started prior to 2007 (when the BRC started)?
6. How would things have been different in this case if the BRC did not exist?
7. Were there any particular challenges that made it difficult to achieve this impact?
8. Who within the OUH would have noticed the impact?
9. Were there also impacts beyond the OUH?
10. Are there examples of other projects that had impacts outside OUH?

**Indirect impacts**

Collaboration and interaction

1. From your own perspective and experience, could you describe what interactions take place between academic researchers and the OUH?
2. Are there examples where interactions have increased the relevance of the research being carried out (for clinicians, policymakers or managers)?
3. What about interactions that increase the willingness of clinicians and policymakers to use results from research?
4. What are the barriers for these interactions? Drivers and enablers?
5. Does the BRC reduce these barriers or support the drivers and enablers?
6. Do you think the BRC could do more to help promote these interactions?
7. Have there been any changes in these interactions over the past 10 years?
8. Who in the OUH would have noticed these changes?

Absorptive capacity, infrastructure and human capital

1. Has there been any equipment or other infrastructure that has become available for or because of the BRC (perhaps mainly for research) that has then become available for wider OUH use? (this could be improvements in care related to conducting a trial)
2. Any other research-related changes or improvements in the setting where care is delivered?
3. Are there clinical or other OUH staff who have become involved in research for BRC work but hadn’t been involved before?
4. If so, have there been effects for them like getting more training, skills or knowledge updating?
5. Do staff become more interested and positive towards research through this involvement?
6. Who in the OUH would have noticed these changes?

Action and participatory research

1. Related to patient involvement in research, does the BRC’s approach to this differ from standard approaches in the OUH?
2. If so, has this had any impacts in the OUH outside BRC projects, for instance on the level of patient engagement in other research activities?
3. If not, do you think approaches should be different and how could this be encouraged to happen?

Economic impacts

1. Are there any examples of work by your Research Theme that have had, or are likely to have, a detectable impact on economic activity in the Oxford region or elsewhere?
2. If so, please tell me what type and magnitude of impact you would expect, and over what approximate time scale: new jobs, new investment, anything else?
3. Who are the key contacts in industry we should approach for their views on these impacts?

In this economic impact section, we want to know what if any impacts you expect your Research Theme (or Working Group) has had and/or will have on economic activity in the Oxford area or more widely.

**Closing**

1. Has the BRC had any other health gain or cost saving impacts beyond the OUH that we have not discussed?
