# Supplementary material for: Does a biomedical research centre affect patient care in local hospitals?
Source: Health Res Policy Syst. 2017 Jan 21;15:2. doi: 10.1186/s12961-016-0163-7 (PMC5251230; doi:10.1186/s12961-016-0163-7)
Supplement: Additional file 2: — Protocol for interviews with clinical staff at Oxford University Hospitals NHS Foundation Trust. (DOC 34 kb) [file 12961_2016_163_MOESM2_ESM.doc]

**Additional file 2:**

**Protocol for interviews with clinical staff at Oxford University Hospitals NHS Foundation Trust**

**Preamble:**

Thank you very much for agreeing to do this interview.

Just to remind you, the purpose of the interview is for us to gather information from you about whether and how research taking place at or by the OUH might be affecting your clinical directorate/division. The study has been commissioned by the NIHR Oxford Biomedical Research Centre (BRC), but we would like to discuss your observations related to research in general, so you don’t need to know anything about the BRC to give us useful information. No advance preparation for the interview is necessary.

In this study, we are interested in different types of impacts that research activity could be having.

One type is direct impacts, where there was a specific research outcome that has been used to improve the process and outcomes of care, or bring down costs in the hospital.

The second type is indirect. These impacts would be less clear-cut ways that research has had an effect. For instance: it could have contributed to staff training or awareness of new treatments or approaches (from whatever source), helped retain quality staff, or helped provide access to infrastructure or equipment outside of research studies.

**Introduction:**

1. What is your role and, briefly, what services and patient groups are covered by your clinical directorate/division?

**Part 1: Observations about research as it relates to the directorate/division’s clinical work**

First, I’d like to ask you some questions to see what sort of role research plays in your clinical work, both today and looking back over the past 10 years or as far back as you feel able to comment.

1. Would you say that research affects the work done by you and the staff in this directorate/division?
   1. If yes: Very briefly (because we will discuss this in more detail later on), what sorts of effects do you have in mind?
2. How much are the clinical staff in your directorate/division involved in research?
   1. Has this changed over time - how/why/evidence?
   2. Who do the staff interact with in their research involvement (Oxford University researchers or others) and has collaboration between your staff and Oxford University changed in degree or qualitatively over the last 10 years?
3. Are there any factors that encourage the staff in your directorate/division to get involved in research?
   1. Any factors that discourage them?
   2. Have these factors changed at all over the past 10 years or so? (how/why?)
   3. Do you have any examples of these changes?
4. Have you observed benefits for patients of your staff being involved in research?
   1. Could you explain what you mean or give any examples?
   2. What about downsides?
   3. Could you explain what you mean or give any examples?
5. Have staff in your directorate/division changed over time in their receptiveness to learning from research, and changing practice accordingly?
6. There are some other ways that research might impact healthcare settings I’d like to ask you about, to see if you have observed any of these in your directorate/division, and if so when they occurred:

Has it…

- 1. Made any infrastructure or equipment available, or improved infrastructure?
  2. Brought in any additional personnel, or training for existing personnel, to do clinical work?
  3. Affected the ways that care is delivered- processes used in health services?
  4. Enabled clinical trials to take place? Or more trials? Or helped them run more effectively?
  5. Helped OUH patients access novel technologies that they wouldn’t have otherwise?
  6. Resulted in more patients going into trials where they get closer monitoring and support than they would otherwise?
  7. Changed the way personnel are organised, in terms of staff having time to do research, or having more evidence-based approaches to how the system is organised?
  8. Encouraged clinicians who don’t do research to become more willing to use research findings and engage with research?
  9. Improved how relevant the research is to policymakers’/ patients/ clinicians?
  10. Raised the profile of research in your directorate/division?

1. Are there any changes that could enable OUH patients and clinicians to benefit more from research being carried out at and with Oxford University?
   - *E.g. how your colleagues get involved, including shaping the research and how it is done?*
   - *E.g. How patients find out research happening and about its results?*

**Part 2: Observations about BRC-specific research activities**

Now, I’d like to ask you some questions about what the BRC does, thinking about today and going back to when the BRC started in 2007.

1. Are you familiar with the BRC?
   1. How have you heard about it/ interacted with it?
   2. How much of the research in your directorate/division involves the BRC?
2. Would you say that the presence of the BRC, has had any effect on the research activities happening in your directorate/division?
   - *E.g. collaboration with the University, contact with industry*
3. What do you think the BRC brings (positive or negative), to the hospital and patient care? Has the BRC been a part of any of the research impacts you have mentioned up to now?
4. To probe that a little further: completely hypothetically, if the BRC were to be discontinued, how would this affect you, your staff and patients? In answering, please consider the way that the BRC works as well as the funding it has for research.
5. Conversely, if the BRC were to double in scale, how if at all would this affect you, your staff and patients?

**Part 3: Specific examples of BRC projects**

1. Are there any BRC projects that have affected the quality or efficiency of patient care delivered in your directorate/division?

Before starting this set of interviews, we looked at documents from the BRC and spoke to leaders of the BRC Research Themes and Working Groups to see what sort of work they have done. You have already mentioned projects A, B, C…. I’d like to ask whether you are familiar with the following additional examples of projects we came across.

1. Are you familiar with project X? If so, could you tell me a bit about:
2. What this project entailed and who was involved
3. How it came about
4. The effects it has had on OUH staff (those who were involved and others), if any
5. The effects it has had on patients, if any
6. Its effects on the hospital’s costs

[INSERT: Sample list to be taken from those that we think would be relevant for each sub-directorate/division]
